# Comparison of analgesic effects of nimesulide, paracetamol, and their combination in animal models

**DOI:** 10.4103/0253-7613.71913

**Published:** 2010-12

**Authors:** Mushtaq Ahmed, Prerna Upadhyaya, Vikas Seth

**Affiliations:** Department of Pharmacology, Mahatma Gandhi Medical College, Sitapura, Jaipur 302 022, India

**Keywords:** Analgesic effects, nimesulide, paracetamol

## Abstract

**Objectives:**

To compare the analgesic activity of nimesulide and paracetamol alone and their combination in animal models for the degree of analgesia and the time course of action.

**Materials and Methods:**

Analgesia was studied in albino rats using formalin test and in albino mice using writhing test and the radiant heat method. For each test, four groups of six animals each were orally fed with a single dose of nimesulide, paracetamol, and combination of nimesulide + paracetamol and gum acacia as control, respectively.

**Results:**

In all the three test models, all three drug treatments showed significant analgesia (*P* < 0.001) as compared to control, but there was no significant difference in the analgesia produced by either drugs alone or in combination. The radiant heat method demonstrated a quicker onset and longer duration of action with nimesulide, whereas writhing test showed a quicker onset of action with paracetamol. In formalin test, greater degree of analgesia was seen with individual drugs than that of the combination, though this difference was not statistically significant.

**Conclusions:**

Nimesulide and paracetamol combination offers no advantage over nimesulide alone or paracetamol alone, either in terms of degree of analgesia or onset of action. Therefore, our study supports the reports claiming irrationality of the fixed dose combination of nimesulide and paracetamol.

## Introduction

Nonsteroidal anti-inflammatory drugs (NSAIDs) are the mainstay of treatment in pain.[1] Various fixed dose combinations of NSAIDs are available in the market, particularly those of paracetamol and ibuprofen. Following this trend, many drug companies have introduced nimesulide and paracetamol combination and claim that it has faster onset and longer duration of analgesic and antipyretic effects than either drugs alone.[2] On the other hand, some authors have claimed that the combination of nimesulide and paracetamol does not offer any such advantage and is therefore irrational.[3] Nimesulide is an NSAID and a preferential COX II inhibitor. It is a potent analgesic, antipyretic, and anti-inflammatory drug.[4,5] In addition, nimesulide does not have significant gastroduodenal side effects.[6] The onset of analgesic and antipyretic action of oral nimesulide is 1.6–3.17 h and duration of action is 10–12 h.[1] Unlike nimesulide, paracetamol is a nonselective COX inhibitor and is an effective analgesic and antipyretic, but a poor anti-inflammatory drug. The onset of action is within 30 min and duration of action is 3–6 h.[1]

Taking these controversial reports into account, this study was designed to evaluate the analgesic activity of nimesulide and paracetamol alone and in combination in experimental animal models in terms of degree of analgesia and time course of action.

## Materials and Methods

### Animals

Healthy albino Wistar rats (3 months old) weighing 150–200 g and albino mice (6- to 7-week-old) weighing 20–40 g were used for antinociceptive tests: formalin test, writhing test, and radiant heat method. Animals were housed in appropriate cages in uniform hygienic conditions and fed with standard pellet diet (Lipton India Laboratories, Bangalore) and water *ad libitum* and were fasted overnight before the day of experiment. Animals were housed within the departmental animal house, and the room temperature was maintained at 27°C. The protocol was approved and carried out after the permission of Institutional Animal Ethics Committee.

### Investigational drugs and dosage preparation

Tablet paracetamol 500 mg (Glaxo-Smithkline, Dr. Annie Besent Road, Worli, Mumbai) was purchased from the hospital pharmacy counter. Tablet nimesulide 100 mg (Emcure Pharmaceuticals Ltd., Dopadi, Pune) was also procured from the same hospital outlet. The appropriate body weight adjusted doses of test drugs as extrapolated from doses used in similar studies conducted previously to be 13.5 mg/200 g rat and 1.45 mg/20 g mouse for paracetamol, and 1.8 mg/200 g rat and 0.26 mg/20 g mouse for nimesulide were used.[7,8]

Formulations were made as suspension prepared in gum acacia 2% w/v uniformly mixed. The formulations were fed to the animals through gastric tube (9 mm) for rat and 2–3 cm polythene tubing sleeved on an 18–20 gauge blunted hypodermic needle for mice. The vehicle gum acacia (2% suspension) alone was used as a control in all the groups.

### Experimental protocol

Animals (*n* = 72) were allocated to three main groups (GI, GII, and GIII) of 24 animals each. GI consisted of 24 rats, and both GII and GIII consisted of 24 mice each. Depending on the treatment design, each group was further divided into four subgroups of six animals each receiving gum acacia as the control (GIc, GIIc, GIIIc, and GIVc), paracetamol (GIp, GIIp, GIIIp, and GIVp), nimesulide (GIn, GIIn, GIIIn, and GIVn), and combination of paracetamol and nimesulide (GIpn, GIIpn, GIIIpn, and GIVpn), respectively.

### Formalin test

Twenty minutes after p.o. administration of control or investigational drugs, all the rats (*n* = 24) in GI received 50 μL of 5% formalin subcutaneously into the plantar portion of left hind paw by using tuberculin syringe to produce chemically induced pain. Each animal was put in a transparent cage made of perspex material and observed for behavioral scoring for 30 min, starting 20 min after formalin injection.[9] Pain severity was graded from g0 to g3 and time spent in each grade was recorded as t0 to t3 using following indices:

1. g0 × t0 (grade 0 for t0 s)= pain free (injected paw not favored)
2. g1 × t1 (grade 1 for t1 s) = mild pain (injected paw raised high on floor)
3. g2 × t2 (grade 2 for t2 s) = moderate pain (injected paw was elevated)
4. g3 × t3 (grade 3 for t3 s) = severe pain (injected paw was licked and bitten or shaken)

Pain score was calculated as:

= g0t0 + g1t1 + g2t2 + g3t3

t0 + t1 +t2 + t3

### Writhing test

Calculated, weight adjusted, doses of test drugs, i.e. paracetamol 5 mg/mL and nimesulide 1 mg/mL in the vehicle, i.e. 2% gum acacia and the control were given p.o. 20 min prior to their antinociceptive effect as tested by counting the number of writhes by calculating the average number of writhes recorded every 30 min up to 240 min for each test group after injection of 1% (1 mL/100 g) acetic acid i.p.[10]

The antinociceptive activity was calculated as percent maximum possible effect (% MPE).

$$\mathrm{Mean}\;\mathrm{writhes}\;\mathrm{GIIc}\;-\;\mathrm{Mean}\;\mathrm{writhes}\;\mathrm{test}\;\%\;\mathrm{MPE}\;=\;\frac{\mathrm{Mean}\;\mathrm{writhes}\;\mathrm{GIIc}}{\mathrm{Test}\;\mathrm{groups}\;=\;\mathrm{GIIp},\;\mathrm{GIIn},\;\mathrm{GIIpn}.}\;\times \;100$$

### Radiant heat method

Prior to subjecting mice to their individual analgesic response to test drugs, animals were subjected to a preliminary screening and mice showing tail flick response in 10 s were selected. Test drugs were administered and antinociceptive response checked as done for the writhing test. Each mouse was restrained, and radiant heat was applied to a portion of tail (about 5 cm from the tip) placed 2 mm above (5A) heating wire of Analgesiometer (INCO). The current was allowed to flow through heating wire and the time taken for the mouse to show tail flick response was recorded every 30 min up to 240 min maximum.[11]

### Statistical analysis

The results of all the three methods namely, formalin test, writhing test, and the radiant heat method are expressed as mean ± SEM. Statistical analysis for the formalin test was done using Wilcoxon–Mann–Whitney test, and data of the other two methods (writhing test and radiant heat method) were statistically analyzed by using ANOVA (two-way classification analysis). A probability value of less than 0.05 (*P* < 0.05) was considered to be statistically significant.

## Results

### Formalin-induced pain

**Figure 1:**
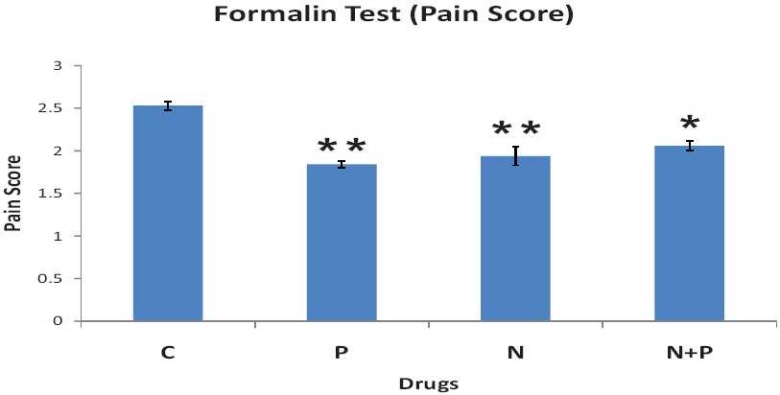
Effect of gum acacia as control (C), nimesulide alone (N), paracetamol alone (P), and nimesulide and paracetamol combination (N + P) on formalin-induced pain in rats. Each point represents mean pain score ± SEM (n = 6). *P < 0.05 and **P < 0.01, compared with control treatment (Wilcoxon– Mann– Whitney test).

Significant analgesia was seen in all three treated groups compared to control [Figure 1]. Analgesia in groups treated with nimesulide and paracetamol alone was greater than that treated with nimesulide and paracetamol in combination. However, this difference was not statistically significant.

### Acetic acid-induced writhing in mice

**Figure 2:**
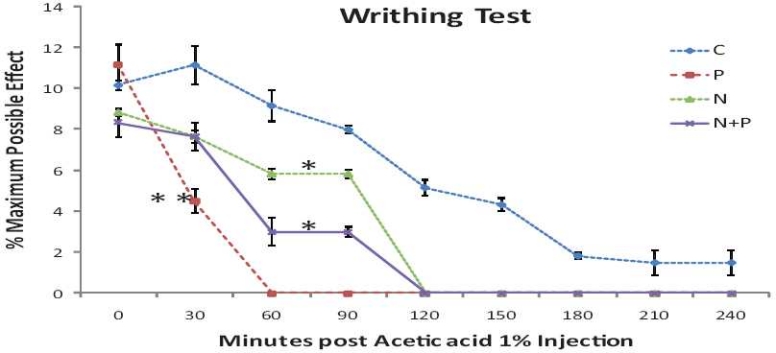
Effect of p.o. administration of nimesulide alone, paracetamol alone, and nimesulide and paracetamol combination on acetic acid (1%) induced writhes in mice. Each point represents mean ± SEM (n = 6). *P < 0.05, **P < 0.01, compared with control treatment (ANOVA by two-way classification analysis).

The drug-treated groups showed significant reduction in number of writhes compared to control [Figure 2]. Studying the time course of action, paracetamol action started within 30 min (less than 50% reduction in number of writhes) with complete abolition of writhes from 60 min onwards. As against this, nimesulide did not reduce the number of writhes to less than 50% even at 60 min and has taken 2 h to completely abolish the writhes. The quicker onset of action of paracetamol has, though not fully, reflected in the combination (N + P) where >50% reduction in writhes has occurred within 60 min. However, this difference in time course of action of the drug treatment was not statistically significant.

### Pain induced by application of heat (radiant heat method) in mice

**Figure 3:**
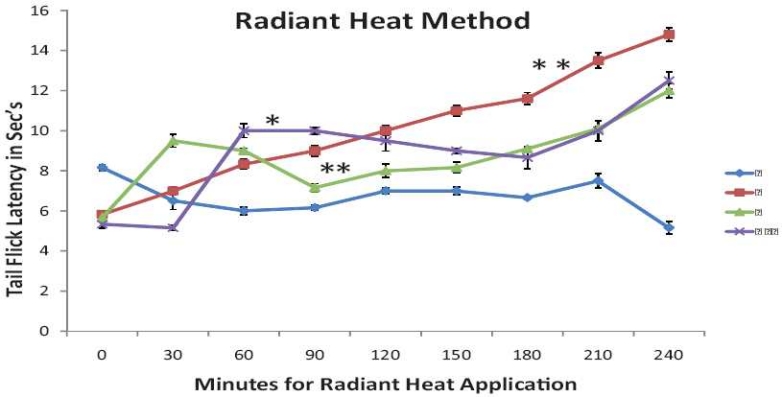
Effect of nimesulide alone, paracetamol alone, and nimesulide and paracetamol combination on radiant heat induced tail flick response in mice. Each point represents mean ± SEM (n = 6). *P < 0.05, **P < 0.01, compared with control (gum acacia) treatment (ANOVA by two-way classification)

The drug treatments have shown significant increase in tail flick latency compared to control [Figure 3]. The onset of action of nimesulide alone was faster than paracetamol alone, showing greater increase in latency of tail flick response at 30 min than paracetamol-treated group. The paracetamol-treated group, however, has shown continuous increase in latency with time when tested up to 240 min. While in nimesulide and nimesulide and paracetamol combination treated groups, latency of response has reduced at 1–1.5 h, again increasing after 2 h. Moreover, the onset of action of nimesulide and paracetamol combination was slower than nimesulide alone, though this difference was statistically insignificant.

## Discussion

This study was undertaken to compare the analgesic efficacy of nimesulide and paracetamol alone and in combination in different animal models. The comparison was done in terms of degree of analgesia and time course of action.

The combination had been introduced in the market on the pretext that it will have the benefits of nimesulide and paracetamol. As paracetamol is faster acting and nimesulide is longer acting, it was presumed that their combination will have additive effects.[2]

However, our study showed that in writhing test, the onset of action of paracetamol was the fastest, whereas in radiant heat method, nimesulide showed the fastest onset of action. Both these methods showed that the combination of nimesulide and paracetamol is definitely slower than either drugs used alone. The formalin test also indicated slightly greater degree of analgesic activity with individual drugs than with their combination. Similar observations have been made by other authors.[3]

Hence, the popular belief that combining the two drugs would give a faster onset does not seem to be substantiated. However, since our observation period was only 240 min, we cannot make any comment about the other advantages of longer duration claimed by the combination. Further studies with longer periods of observation would probably throw more light on the validity of this combination. In addition, pain is a symptom with substantial subjective component as well. It is, therefore, difficult to comment on the effectiveness of an analgesic purely on the basis of animal studies. Hence, it would also be necessary to test the combination on human subjects, both on experimental pain in healthy volunteers as well as clinical pain, before commenting on the appropriateness of this combination. Many studies have compared the analgesic efficacy of nimesulide and paracetamol with other NSAIDs;[12–14] however, there is no such study, which compares individual drugs with the combination. Usually, the fixed dose combinations are introduced in the market to generate prescriptions and make profit with no consideration of the rationality.[15] Combinations of analgesics are more effective if they act through different analgesic mechanisms and act synergistically.[16] In contrast, the components of the fixed dose combination of nimesulide and paracetamol act by the same mechanism of inhibition of prostaglandin biosynthesis. The most important concern with irrational fixed dose combinations is that they expose patients to unnecessary adverse effects.[17] It has been seen that nimesulide is as good analgesic as any other NSAIDs, but its combination with paracetamol increases its hepatotoxic potential.[18] Usage of many available fixed dose combinations is controversial, and it is the need of hour to sensitize all practitioners and consumers against this practice.

## Conclusion

Our study shows that nimesulide alone and paracetamol alone are more efficacious analgesics as compared to the combination of nimesulide and paracetamol. Neither of the drugs potentiates nor synergizes the action of the other. Therefore, there is no pharmacological rationale for the combined administration of nimesulide and paracetamol. However, further studies with longer periods of observation are warranted to evaluate the validity of the combination. In addition, studies of the combination on human subjects are necessary to assess the subjective degree of pain and appropriateness of the combination.
